# Genomic regulation of Krüppel-like-factor family members by corticosteroid receptors in the rat brain

**DOI:** 10.1016/j.ynstr.2023.100532

**Published:** 2023-03-07

**Authors:** Clare L.M. Kennedy, Emily M. Price, Karen R. Mifsud, Silvia Salatino, Eshita Sharma, Simon Engledow, John Broxholme, Hannah M. Goss, Johannes M.H.M. Reul

**Affiliations:** a Neuro-Epigenetics Research Group, University of Bristol, Dorothy Hodgkin Building, Whitson Street, Bristol, BS1 3NY, United Kingdom; b Wellcome Centre for Human Genetics, University of Oxford, Roosevelt Drive, Oxford, OX3 7BN, United Kingdom

## Abstract

Hippocampal mineralocorticoid receptors (MRs) and glucocorticoid receptors (GRs) mediate glucocorticoid hormone (GC) action in the hippocampus. These receptors bind to glucocorticoid responsive elements (GREs) within target genes, eliciting transcriptional effects in response to stress and circadian variation. Until recently, little was known about the genome-wide targets of hippocampal MRs and GRs under physiological conditions. Following on from our genome-wide MR and GR ChIP-seq and Ribo-Zero RNA-seq studies on rat hippocampus, we investigated the Krüppel-like factors (KLFs) as targets of MRs and GRs throughout the brain under circadian variation and after acute stress. In particular, *Klf2, Klf9* and *Klf15* are known to be stress and/or GC responsive and play a role in neurobiological processes including synaptic plasticity and neuronal differentiation. We found increased binding of MR and GR to GREs within *Klf2, Klf9* and *Klf15* in the hippocampus, amygdala, prefrontal cortex, and neocortex after acute stress and resulting from circadian variation, which was accompanied by upregulation of corresponding hnRNA and mRNA levels. Adrenalectomy abolished transcriptional upregulation of specific *Klf* genes. These results show that MRs and GRs regulate *Klf* gene expression throughout the brain following exposure to acute stress or in response to circadian variation, likely alongside other transcription factors.

## 1 Introduction

Glucocorticoid (GC) hormones comprise a group of steroids that regulate critically important physiological functions such as metabolism, immune system, neuroendocrine control, and many central nervous system (CNS) functions (Munck et al., 1984; McEwen, 2012; Reul et al., 2015). In particular, GCs act upon the brain and are thought to facilitate the generation of molecular and cellular responses to stress, supporting behavioural adaptation by promoting long-term memory formation (Reul, 2014; Reul et al., 2015; Mifsud and Reul, 2018). The importance of GC hormone activity in the proper functioning of the CNS is underscored by the wide range of stress-related mental health disorders, including major depression, bipolar disorder and post-traumatic stress disorder (PTSD), in which aberrant GC hormone secretion and function is typically observed (Holsboer, 2000). These disorders affect millions of people, yet the available treatments are often ineffective, most likely due to the poor understanding of the molecular events that underlie their pathophysiology. Elucidating the effects of GC hormones on the CNS may be crucial to delineate the pathophysiology of these mental health disorders, thus contributing to the development of more effective treatments.

As it is already well-established that DNA-binding MRs and GRs mediate many effects of GCs in the brain (Reul and de Kloet, 1985; Mifsud and Reul, 2016), identification of the genomic targets of these receptors should be the next step in unravelling the intricacies of GC hormone action. Recently, using chromatin immunoprecipitation-sequencing (ChIP-seq), we performed a comprehensive study to identify the genomic regions that undergo MR and GR interaction in the rat hippocampus under GC-relevant physiological conditions including circadian variation and acute stress (Mifsud et al., 2021). Moreover, we conducted Ribo-Zero-RNA-seq to assess changes in both intronic and exonic RNA transcript levels under these physiological conditions (Mifsud et al., 2021). We thoroughly examined our ChIP- and RNA-seq data to identify genomic targets of MRs and GRs warranting further investigation. Several Krüppel-like-factor (KLF) family members emerged as prominent genes in the sequencing data, as well as downstream in motif and pathway analyses (Mifsud et al., 2021). In particular, *Klf2, Klf9* and *Klf15* all showed significantly increased binding of MRs and GRs to glucocorticoid response elements (GREs) after acute stress and during circadian changes; genomic events which were accompanied by rises in RNA levels of the corresponding genes (Mifsud et al., 2021). Moreover, several *Klf* genes, such as *Klf4*, displayed a transcriptional response after acute stress or during circadian changes, however, in the absence of any MR or GR binding. Members of the KLF family are abundantly expressed in the brain, where they regulate processes such as neural development and repair after brain injury (Yin et al., 2015). In particular, KLFs highlighted by our sequencing studies have been linked to blood brain barrier function (Shi et al., 2013), neurogenesis (Qin and Zhang, 2012), synaptic plasticity (Scobie et al., 2009) and neuronal differentiation and migration (Ohtsuka et al., 2011). Several studies have examined KLFs in response to stress (Datson et al., 2011; Reddy et al., 2009), however, the role of MRs and GRs in the expression of these transcription factors (TFs) has not been fully investigated.

Therefore, further to our MR and GR ChIP-seq and RNA-seq studies (Mifsud et al., 2021), we here present ChIP-qPCR and RNA experiments on the stress- and/or circadian-responsive KLFs in the rat hippocampus as well as other stress-sensitive brain regions such as the amygdala, prefrontal cortex and neocortex (Kolber et al., 2008; Shepard et al., 2003; Mizoguchi et al., 2004). In addition, we used a surgical intervention strategy to ascertain the role of MR and GR in stress-induced changes in KLF gene transcription.

## 2 Methods

### 2.1 Animals

All experimental animal work was approved by the University of Bristol Ethical Committee and the Home Office of the United Kingdom (Animal Scientific Procedures Act, 1986; UK). Male Wistar rats (150–175 g) were purchased from Envigo (Oxon, UK) and group-housed (two to three animals per cage). Animals were kept under standard light (lights on 5:00–19:00 h; 80–100 Lux) and environmentally controlled conditions (temperature 21 ± 1 °C; relative humidity 40–60%) with food and water available *ad libitum*. Until the day of the experiment all rats were handled (2 min per rat per day; min. 5 days) to reduce any nonspecific stress effects. Independent cohorts of rats were used for the sequencing and validation studies.

### 2.2 Animal experiments

#### 2.2.1 Sequencing validation and brain-wide investigations

The ChIP-seq and RNA-seq data on *Klf* genes were extracted from the databases first presented in Mifsud et al. (2021). In the (original) sequencing study and present validation and other studies, baseline rats were killed straight from their home-cages between either 7–9am (circadian trough, baseline AM (BLAM)) or 5–7pm (circadian peak, baseline PM (BLPM)). Alternatively, rats were killed 30 min (FS30), 60 min (FS60) or 120 min (FS120) after the start of a forced swim (FS) challenge (15 min in 25 ± 1 °C-water).

#### 2.2.2 Adrenalectomy experiments

For these experiments, rats (6 per group) underwent adrenalectomy (ADX), sham surgery (sham-ADX) or were left undisturbed (‘intact’). Surgeries were performed under isoflurane anaesthesia. Sham surgeries were identical to ADX except that the adrenals were not removed. ADX rats were provided with 0.9% saline containing CORT supplementation (15 mg/l) in their drinking water for 1 week following surgery. One-week post-surgery, CORT supplementation was discontinued 1 day before experimentation to clear the body of glucocorticoid hormone.

#### 2.2.3 ChIP-qPCR

Hippocampus or amygdala tissues of two rats were pooled and cross-linked in a 1% formaldehyde buffered solution containing inhibitors, sonicated and chromatin extracted as described previously (Mifsud et al., 2021). Prefrontal cortex and neocortex tissue were used from individual animals. Chromatin (200 μl) was immunoprecipitated after incubation with the following antibodies: anti-MR (MR ab64457; Abcam, Cambridge, UK) or anti-GR (GR 24050-1-AP antibody; Proteintech, Manchester, UK). Antibody specificity data was confirmed by pre-absorption tests and Western blot analysis as previously described (Mifsud et al., 2021). Input samples were prepared from 20 μl of the original chromatin samples by reversing crosslinks (addition of NaCl, final conc. 200 mM, at 65 °C overnight), treatment with RNase A (60 μg/ml, 1 h at 37 °C) and proteinase K (250 μg/ml, overnight at 37 °C). DNA was purified using Qiagen PCR purification kit as per manufacturers’ instructions (Qiagen, Germany). All samples (bounds and inputs) were diluted to a standardized concentration with nuclease-free water for analysis by qPCR using primers/probes listed in Supplementary Table 1. Primers were designed to rat genome reference version Rnor_6.0. Data (i.e., enrichment) are expressed as quantity of bound DNA divided by the respective quantity of input DNA (i.e., B/I), which is a measure of the enrichment of steroid receptor bound to specific genomic sequences.

#### 2.2.4 RNA-RT-qPCR

Each biological sample comprised RNA from hippocampus, amygdala, neocortex, or prefrontal cortex tissue of one rat. Total RNA (1000 ng) was reverse transcribed into cDNA using the QuantiTect Reverse Transcription Kit (Qiagen, UK) as per manufacturer's instructions (15 min at 42 °C; 5 min at 95 °C, BioRad T1000 thermocycler). cDNA was diluted 4-fold in Tris-EDTA and subjected to qPCR analysis using the Taqman primers and FAM/TAMRA probes included in Supplementary Table 2. Expression levels of hnRNA and mRNA in each sample were calculated based on the Pfaffl method of relative quantification (Pfaffl, 2001), standardized to the expression of the house-keeping genes listed in Supplementary Table 3 and expressed as fold-change over BLAM levels. All primers were designed to Rnor_6.0.

### 2.3 Statistical analysis

ChIP-seq and RNA-seq data were analysed with “DiffBind” and “EdgeR” packages, respectively, as previously described (Mifsud et al., 2021). ChIP-qPCR and RNA-RT-qPCR data were statistically analysed using GraphPad Prism software 7.04 (San Diego, CA, USA). Results are presented as group means ± SEM; sample sizes are indicated in the figure legends. Statistical comparisons on normally distributed data were conducted with one-way ANOVA, or two-way ANOVA, and if significant, a Dunnett's or Sidak's *post-hoc* test was performed. Non-parametric data were tested with a Kruskal-Wallis test, and if significant, a Dunn's *post-hoc test* was performed. Statistical results are provided in the figure legends. *P* < 0.05 was considered statistically significant.

#### 2.3.1 Motif analysis

The motif analysis data were generated using Find Individual Motif Occurrence (FIMO) analysis as previously described (Mifsud et al., 2021).

#### 2.3.2 Pathway analysis

Ingenuity Pathway Analysis (IPA) software was used to perform pathway analysis of data from ChIP-seq and RNA-seq experiments (Qiagen, UK) (Kramer et al., 2014). A “Core Analysis” was performed on the data generated as previously described (Mifsud et al., 2021).

## 3 Results

Specific KLF family members are prominent targets of GC hormones following analysis of ChIP- and RNA-sequencing data

**Fig. 1:**
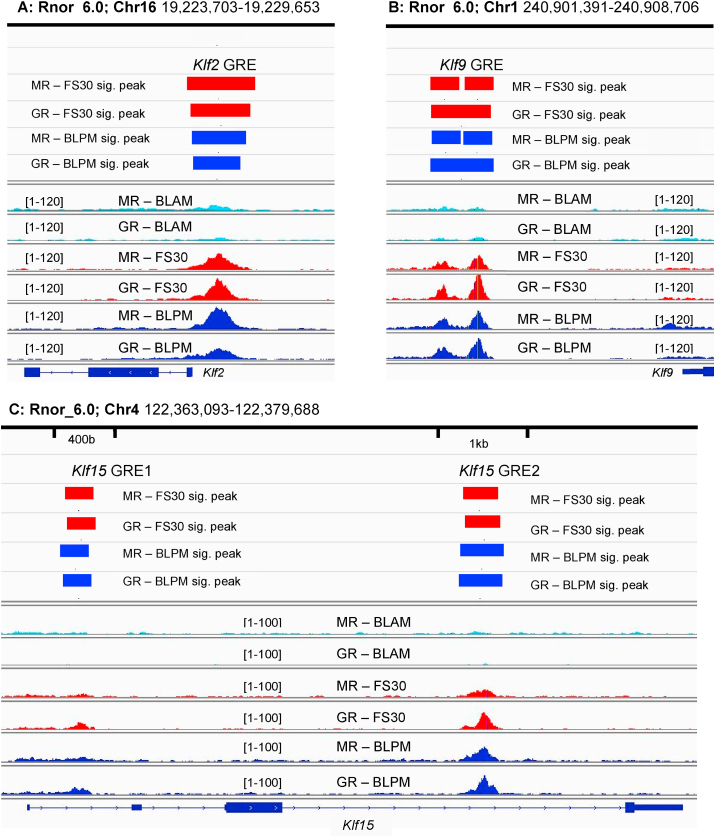
Hippocampal ChIP-seq reveals MR and GR peaks within *Klf2, Klf9* and *Klf15* under baseline and stress conditions. Rats were killed under early morning (BLAM) or late afternoon (BLPM) baseline conditions or 30 min (FS30) after the start of FS (15 min, 25 °C water). Hippocampal tissue underwent ChIP-seq. Genomic loci significantly bound by MRs and GRs were identified by Peak calling (MACS2, FDR<0.05). A, B and C show Integrative Genome Viewer (IGV) images of MR and GR peaks within *Klf2, Klf9* and *Klf15,* respectively*,* at BLAM, FS30 and BLPM. Significant peaks (i.e. above input level) are indicated by the presence of a horizontal red (FS30) or blue (BLPM) bar. Note that there are no significant peaks within these genes under BLAM conditions. Numbers between square brackets indicate the y-axis range. Rnor_6.0, *Rattus norvegicus* genome version 6.0.

Data analysis of ChIP- and RNA-seq data of rat hippocampus tissue was performed (Mifsud et al., 2021) to identify GC-target genes for further investigation. Upon examination of the ChIP-seq data, it was apparent that a number of *Klf* gene family members exhibited significant MR and GR binding peaks under physiological conditions known to show elevated circulating GC levels. These *Klf* genes include *Klf2, Klf9* and *Klf15,* whose MR and GR peaks were found within promoter (*Klf2* and *Klf9)* and intronic regions (*Klf15*) (Fig. 1). The sequence alignment files show very little binding of MRs and GRs under BLAM conditions, but significant receptor binding peaks were seen under FS30 and BLPM conditions (Fig. 1).

Differential binding analysis (DiffBind, FDR<0.05) on these MR and GR ChIP-seq data (peak coordinates can be found in Supplementary Table 4) showed that the binding of both receptors to the hippocampal genome was significantly elevated after acute stress and circadian changes compared to BLAM conditions (Supplementary Fig. 1). MR and GR binding was significantly higher at almost all GREs examined, with the exception of MR binding to *Klf15* GRE1 under BLPM conditions (Supplementary Fig. 1E). Subsequent examination of differential RNA expression analysis revealed that increased receptor binding coincided with a time-dependent increase in intronic and exonic RNA (inRNA and exRNA, respectively) counts of these genes (Supplementary Fig. 2)*.* The significant rise in inRNA and exRNA counts of *Klf2* was seen at the FS30 and FS60 timepoints after which counts returned to baseline levels, rising again at BLPM. The response in *Klf9* and *Klf1*5 RNA occurred across almost all FS timepoints examined and under BLPM conditions. *Klf4,* a gene not bound by MR or GR, responded to stress in a similar fashion to *Klf2,* with elevated levels of RNA counts at FS30, FS60 and FS360.

MR and GR peaks within specific *Klf* genes contain GREs alongside other prominent transcription factor binding motifs

As previously reported (Mifsud et al., 2021), a relatively high proportion (68–70%) of the MR and GR peaks identified by ChIP-seq contained KLF binding motifs alongside glucocorticoid response elements (GREs). Closer inspection of our FIMO analysis outputs (Mifsud et al., 2021) revealed the presence of GREs in all MR and GR peaks within *Klf2, Klf9* and *Klf15*, in addition to other prominent transcription factor binding sites such as early growth response (EGR) motifs, specificity protein (SP) motifs, cAMP-response element binding (CREB) motifs and KLF motifs (Supplementary Tables 5 and 6). Possibly, other transcription factors, including KLFs themselves, bind alongside MRs and GRs to exert transcriptional regulation on these genes during the acute stress response and the circadian drive.

Validation of ChIP- and RNA-seq data in a separate cohort of rats: confirmation of specific *Klf* genes as hippocampal GC target genes

**Fig. 2:**
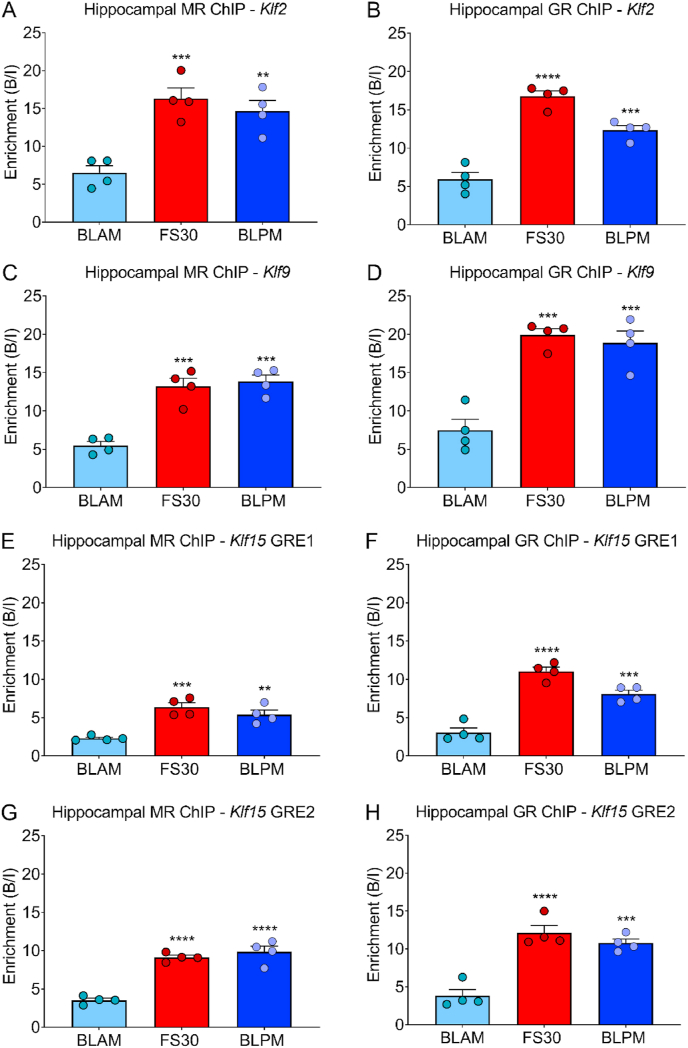
Hippocampal MR and GR binding at GREs within *Klf2 Klf9* and *Klf15* under baseline and stressed conditions. Rats were killed under early morning (BLAM) or late afternoon (BLPM) baseline conditions or 30 min (FS30) after the start of FS (15 min, 25 °C water). Graphs show enrichment of MR and GR, expressed as bound/input (B/I; mean ± SEM, n = 4 per group), at GREs within *Klf2* (A and B), *Klf9* (C and D), *Klf15* (E-H) as determined by MR and GR ChIP-qPCR. Statistical analysis; One-way ANOVA (A) F_(2, 9)_ = 17.14, *p* = 0.0009, (B) F_(2, 9)_ = 55.18, *p* < 0.0001, (C) F_(2, 9)_ = 30.22, *p* < 0.001, (D) F_(2, 9)_ = 28.05, *p* < 0.001, (E) F_(2, 9)_ = 19.62, *p* = 0.0005, (F) F_(2, 9)_ = 52.65, *p* < 0.0001, (G) F_(2, 9)_ = 50.43, *p<*0.0001, (H) F_(2, 9)_ = 31.39, *p* < 0.0001. Dunnett's post hoc test, **P < 0.01, ***P < 0.001, ****P < 0.0001 significantly different from BLAM.

**Fig. 3:**
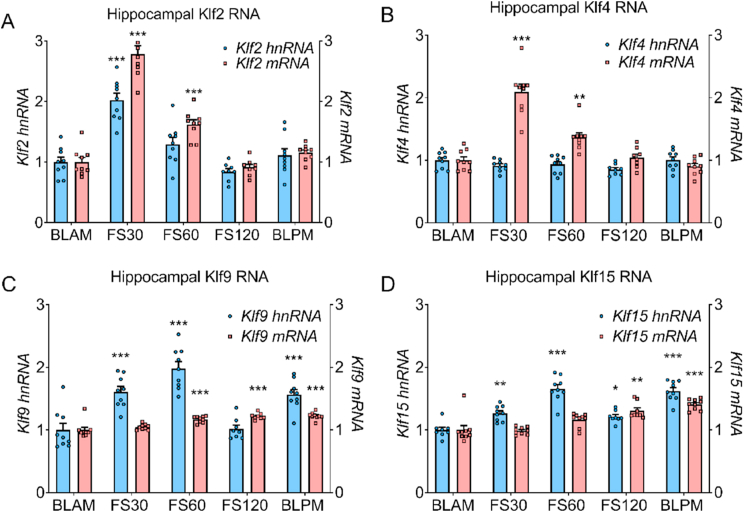
Hippocampal hnRNA and mRNA of *Klf* genes under baseline conditions and following stress. Rats were killed under early morning (BLAM) or late afternoon (BLPM) baseline conditions or 30 min (FS30), 60 min (FS60) or 120 min (FS120) min after the start of FS (15 min, 25 °C water). Graphs show hnRNA (blue bars) and mRNA (red bars) levels of *Klf2* (A), *Klf4* (B) *Klf9* (C) and *Klf15* (D). Data are shown as relative RNA copy number calculated using the Pfaffl method of analysis, standardised to the expression of the house keeping genes *Hprt1* and *Ywhaz* (mean ± SEM, n = 8–9 per group). Statistical analysis: one-way ANOVA; (A) *Klf2* hnRNA F_(4, 39)_ = 21.91, *p* < 0.0001, *Klf2* mRNA F_(4, 39)_ = 81.88, *p* < 0.0001, (B) *Klf4* hnRNA F_(4, 39)_ = 2.178, *p* = 0.0895, *Klf4* mRNA F_(4, 39)_ = 41.14, *p* < 0.0001, (C) *Klf9* hnRNA F_(4, 39)_ = 21.38, *p* < 0.0001, *Klf9* mRNA F_(4, 39)_ = 16.7, *p* < 0.0001, (D) *Klf1*5 hnRNA F_(4, 39)_ = 31.41, *p* < 0.0001. Dunnett's post hoc test, *P < 0.05, **P < 0.01, ***P < 0.001, compared with BLAM group. Kruskal-Wallis; (D) *Klf1*5 mRNA χ^2^ (4) = 27.72, *p* < 0.0001. Dunn's post hoc test, **P < 0.01, ***P < 0.001 significantly different from BLAM.

To validate the findings of ChIP- and RNA-seq experiments regarding KLFs and other genomic targets, ChIP and RNA analyses were conducted on a separate cohort of rats which had been killed under identical conditions (BLAM, FS30, BLPM). The consistency of our experimentations is reflected by the highly similar plasma CORT profiles as reported previously (Mifsud et al., 2021). ChIP-qPCR and RNA-qPCR was performed to assess the binding of hippocampal MRs and GRs to GREs within KLF genes and the associated transcriptional responses. Results of ChIP- and RNA-qPCR experiments on hippocampus (Fig. 2, Fig. 3) closely mirrored those of the ChIP- and RNA-seq studies on this tissue (Supplementary Figs. 1 and 2). Consistent with our data in the sequencing study, ChIP-qPCR showed that MR and GR binding significantly increased at GREs within *Klf2, Klf9* and *Klf15* under both FS and BLPM conditions (Fig. 2). Furthermore, consistent with results shown by our RNA-seq, FS resulted in transcriptional activation of *Klf2*, *Klf4, Klf9* and *Klf15* in the hippocampus (Fig. 3), while *Klf9* and *Klf15* exhibited transcriptional responses to BLPM conditions as well. Hence, hnRNA and mRNA levels measured by RNA-qPCR corresponded with the intronic and exonic RNA count data determined by RNA-seq (Mifsud et al., 2021). These findings highlight the robustness and high quality of the ChIP- and RNA-seq datasets.

Pathway analysis of GC target genes predicts *Klf* genes as important transcriptional regulators with roles in neurobiological processes

Pathway analysis predicted a number of KLFs to act as upstream regulators of genes which are bound by MRs and GRs in response to rises in GC secretion (Supplementary Table 7). This prediction, combined with findings from motif analysis, indicates that many transcriptional targets are shared by MRs, GRs and KLFs. Pathway analysis of ChIP- and RNA-seq datasets predicted a role of *Klf9* and *Klf15* in neurobiological functions, such as neuronal development and synaptic plasticity and in behavioural processes such as learning, cognition and anxiety (Supplementary Table 8).

### 3.1 MR and GR regulation of *Klf* genes occurs in the amygdala and cortical brain regions

**Fig. 4:**
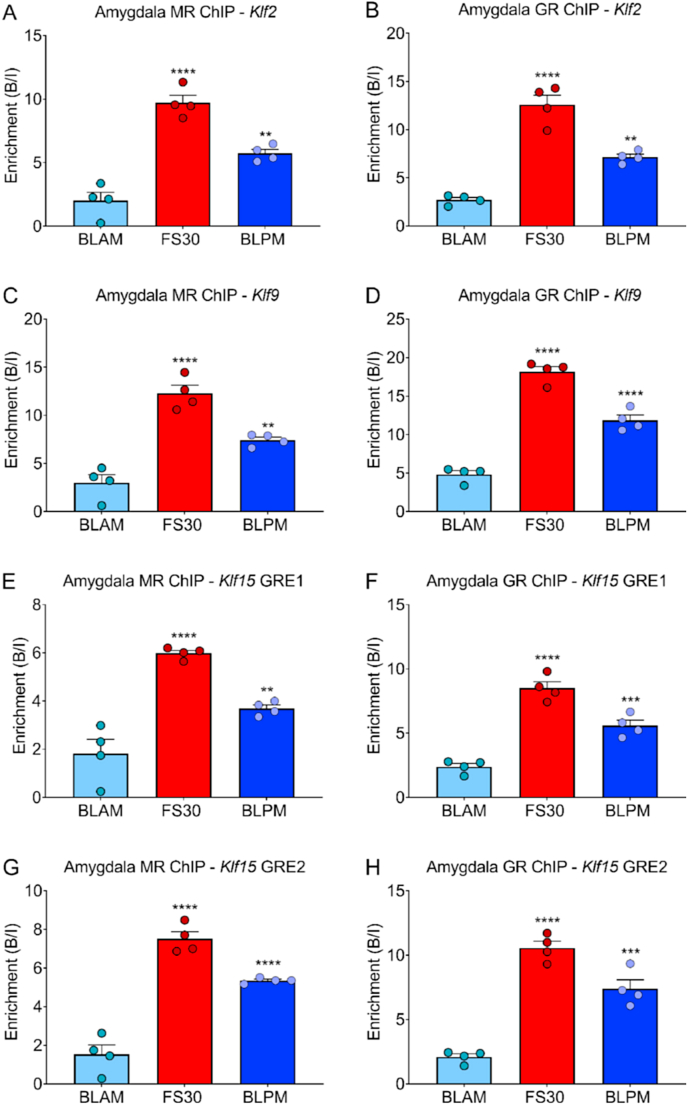
Amygdala MR and GR binding at GREs within *Klf2, Klf9* and *Klf15* under baseline and stressed conditions. Rats were killed under early morning (BLAM) or late afternoon (BLPM) baseline conditions or 30 min (FS30) after the start of FS (15 min, 25 °C water). Graphs show enrichment of MR and GR, expressed as bound/input (mean ± SEM, n = 4 per group), at GREs within *Klf2* (A and B), *Klf9* (C and D), *Klf15* (E-H) as determined by MR and GR ChIP-qPCR. Statistical analysis; One-way ANOVA (A) F_(2, 9)_ = 51.49, *p* < 0.0001, (B) F_(2, 9)_ = 63.46, *p* < 0.0001, (C) F_(2, 9)_ = 42.92, *p* < 0.0001, (D) F_(2, 9)_ = 114, *p* < 0.0001, (E) F_(2, 9)_ = 34.73, *p* < 0.0001, (F) F_(2, 9)_ = 56.24, *p* < 0.0001, (G) F_(2, 9)_ = 72.83, *p* < 0.0001, (H) F_(2, 9)_ = 68.58, *p* < 0.0001. Dunnett's post hoc test, **P < 0.01, ***P < 0.001, ****P < 0.0001 significantly different from BLAM.

**Fig. 5:**
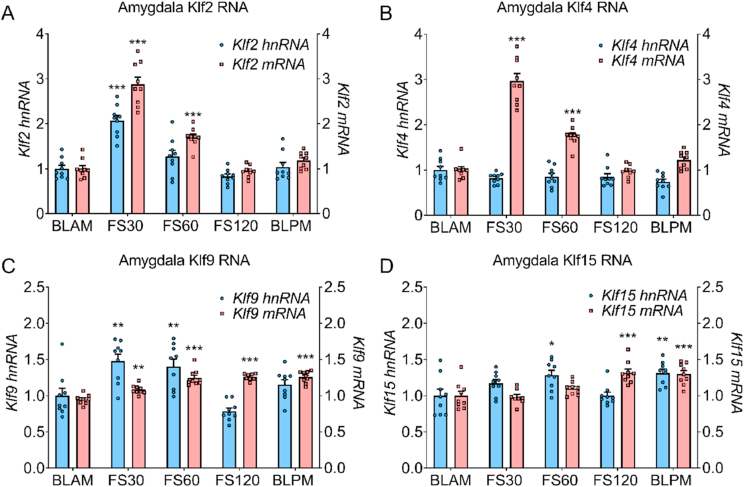
Amygdala hnRNA and mRNA of KLFs under baseline conditions and following stress. Rats were killed under early morning (BLAM) or late afternoon (BLPM) baseline conditions or 30 min (FS30), 60 min (FS60) or 120 min (FS120) min after the start of FS (15 min, 25 °C water). Graphs show hnRNA (blue bars) and mRNA (red bars) levels of *Klf2* (A), *Klf4* (B) *Klf9* (C) and *Klf15* (D). Data are shown as relative RNA copy number calculated using the Pfaffl method of analysis, standardised to the expression of the house keeping genes *Hprt1* and *Gapdh* (mean ± SEM, n = 8–9 per group). Statistical analysis: one-way ANOVA; (A) *Klf2* hnRNA F_(4, 40)_ = 23.36, *p* < 0.0001, *Klf2* mRNA F_(4, 40)_ = 79.11, *p* < 0.0001, (B) *Klf4* hnRNA F_(4, 40)_ = 2.04, *p* = 0.35, *Klf4* mRNA F_(4, 40)_ = 78.69, *p* < 0.0001, (C) *Klf9* hnRNA F_(4, 40)_ = 11.07, *p* < 0.0001, *Klf9* mRNA F_(4, 40)_ = 26.97, *p* < 0.0001, (D) *Klf1*5 hnRNA F_(4, 40)_ = 5.493, *p* = 0.00131, *Klf1*5 mRNA F_(4, 39)_ = 11.32, *p* < 0.0001. Dunnett's post hoc test, *P < 0.05, **P < 0.01, ***P < 0.001 significantly different from BLAM.

As MRs and GRs are not exclusively located in the hippocampus and other brain regions, like the amygdala, prefrontal cortex (PFC) and neocortex (NEO), are known to be affected by GC hormones, we investigated these extra-hippocampal brain regions with MR and GR ChIP-qPCR as well. For the first time, we present data showing MR and GR binding to *Klf* GREs and the associated transcriptional responses of these genes in these brain regions following stress and circadian changes. Fig. 4, Fig. 5 show ChIP- and RNA-qPCR data for the amygdala, respectively, while our findings on the PFC and NEO can be found in Supplementary Figs. 3–6. Similar to the hippocampus, MR and GR binding to GREs within *Klf2, Klf9* and *Klf15* and corresponding RNA levels increased significantly within the amygdala (Fig. 5), PFC (Supplementary Fig. 5) and NEO (Supplementary Fig. 6) after acute stress and/or BLPM conditions. *Klf4* mRNA levels, but not hnRNA levels, rose significantly after FS within the amygdala (Fig. 5B), PFC (Supplementary Fig. 5B) and NEO (Supplementary Fig. 6B). In PFC, FS resulted in significantly increased levels of both *Klf4* hnRNA and mRNA (Supplementary Fig. 5B). Under BLPM conditions, *Klf4* mRNA levels increased in the prefrontal cortex (Supplementary Fig. 5B). Comparing hnRNA and mRNA responses, we observed that changes in Klf2 transcripts were very similar across the four examined tissues (Fig. 3, Fig. 5A, Supplementary Figs. 5A and 6A). Regarding the other Klf genes, there were slight differences between stress- and BLPM-related responses in hnRNA and mRNA (Fig. 3C and D, 5C-D, Supplementary Figs. 5B–D, 6C-D).

Adrenalectomy attenuates the FS-induced transcriptional response of *Klf2,* while the rise in *Klf9* and *Klf1*5 RNA is completely abolished

**Fig. 6:**
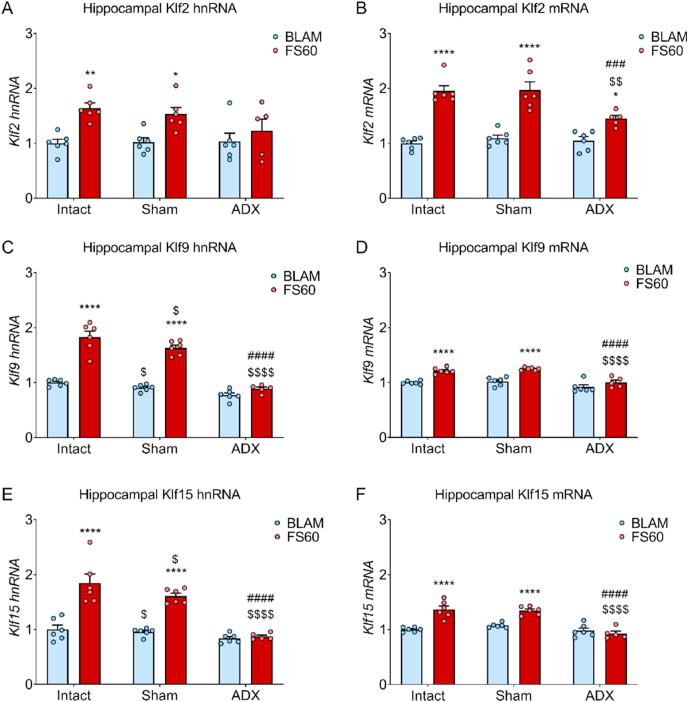
The effect of ADX on hippocampal hnRNA expression of KLF genes under BLAM conditions and following acute FS stress. Rats underwent sham surgery or adrenalectomy (ADX) and 1 week later, alongside an intact group, were killed under BLAM conditions (∼9:00 a.m.) or 60 min after the onset of FS (15 min, 25 °C water). Graphs show hnRNA levels of *Klf2* (A), *Klf9* (C) and *Klf15* (E) and mRNA levels of *Klf2* (B), *Klf9* (D) and *Klf15* (F).. Data are shown as relative RNA copy number calculated using the Pfaffl method of analysis, standardised to the expression of the house keeping genes *Hprt1* and *Ywhaz* (mean ± SEM, n = 5–6 per group). Statistical analysis: two-way ANOVA; (A) effect of surgery: F_(2, 29)_ = 1.215, *p* = 0.311, effect of stress: F_(1, 29)_ = 19.6, *p* < 0.001, interaction surgery x stress: F_(2, 29)_ = 1.655, *p* = 0.209, (B) effect of surgery: F_(2, 29)_ = 5.624, *p* = 0.0087, effect of stress: F_(1, 29)_ = 107.4, *p* < 0.0001, interaction surgery x stress: F_(2, 29)_ = 5.615, *p* < 0.01, (C) effect of surgery: F_(2, 29)_ = 57.44, *p* < 0.0001, effect of stress: F_(1, 29)_ = 148.5, *p* < 0.0001, interaction surgery x stress: F_(2, 29)_ = 23.09, *p* < 0.0001, (D) effect of surgery: F_(2, 29)_ = 21.59, *p* < 0.0001, effect of stress: F_(1, 29)_ = 58.54, *p* < 0.0001, interaction surgery x stress: F_(2, 29)_ = 3.738, *p* = 0.0359, (E) effect of surgery: F_(2, 29)_ = 24.17, *p* < 0.0001, effect of stress: F_(1, 29)_ = 55.88, *p* < 0.0001, interaction surgery x stress: F_(2, 29)_ = 12.37, *p* = 0.0001, (F) effect of surgery: F_(2, 29)_ = 23.71, *p* < 0.0001, effect of stress: F _(1, 29)_ = 36.28, *p* < 0.0001, interaction surgery x stress: F_(2, 29)_ = 14.66, *p* < 0.0001, Sidak's post hoc tests, *P < 0.05, **P < 0.01, ****P < 0.0001 significantly different from BLAM within the same surgery group. $ *P* < 0.05, $$ *P* < 0.01, $$$$ *P* < 0.0001 significantly different from intact within same treatment group, ###*P* < 0.001*,* ####*P* < 0.0001 significantly different from sham within the same treatment group.

To examine the effects of the complete removal of endogenous CORT on downstream transcriptional responses following acute stress, hnRNA and mRNA levels of *Klfs* were examined in hippocampus tissue of ADX rats collected in a previous study (Kennedy et al., 2020). Plasma CORT levels were shown to be below detection levels in ADX rats compared to the sham and intact groups (Kennedy et al., 2020). ADX abolished the FS-induced rise in *Klf2*, *Klf9* and *Klf1*5 hnRNA (Fig. 6A–C) and *Klf9* and *Klf1*5 mRNA (Fig. 6B and C), and attenuated the rise in mRNA levels of *Klf2* (Fig. 6B).

ADX has no effect on the transcriptional response of the non-MR- and non-GR-bound gene *Klf4*

**Fig. 7:**
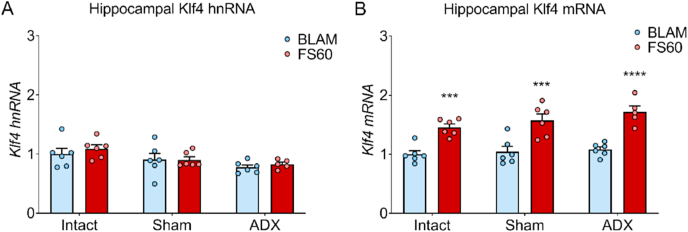
Adrenalectomy does not affect the stress-induced rise in RNA levels of the non-MR and non-GR target gene *Klf4*. Rats underwent sham surgery or adrenalectomy (ADX) and 1 week later, alongside an intact group, were killed under BLAM conditions (∼9:00 a.m.) or 60 min after the onset of FS (15 min, 25 °C water). mRNA levels of *Klf4* are shown as relative RNA copy number calculated using the Pfaffl method of analysis, standardised to the expression of the house keeping genes Hprt1 and Ywhaz (mean ± SEM, n = 5–6 per group). Statistical analysis: two-way ANOVA; (A) effect of surgery: F_(2, 29)_ = 5.529, *p* = 0.0092, effect of stress: F_(1, 29)_ = 0.620, *p* = 0.438, interaction surgery x stress: F_(2, 29)_ = 0.211, *p* = 0.810, (B) effect of surgery: F_(2, 29)_ = 2.304, *p* = 0.118, effect of stress: F_(1, 29)_ = 69.78, *p* < 0.0001, interaction surgery x stress: F_(2, 29)_ = 0.663, *p* = 0.523. *P < 0.05, ***P<0.001, ****P < 0.0001 significantly different from BLAM within the same drug/or group.

RNA levels of *Klf4* were examined as a negative control for GC involvement in stress-evoked responses, to confirm that ADX did not lead to any effects on the regulation of a *Klf* gene that is not targeted by MR or GR binding. ADX had no effect on the mRNA levels of *Klf4* (Fig. 7), while an effect of FS stress remained.

## 4 Discussion

Here, we present a series of experiments on KLF transcription factors using a combination of molecular and surgical approaches. Together these data extend the findings of our sequencing experiments and subsequent *in silico* bioinformatics analyses that indicates a link between the KLF family of transcription factors and genomically acting corticosteroid receptors. The findings of our hippocampal ChIP-qPCR and RNA-qPCR experiments further contribute to the robust validation of our sequencing experiments as described by Mifsud et al. (2021). Intriguingly, pathway analysis of receptor binding (ChIP) datasets highlighted a role of KLFs in cell-biological aspects of neurobiological processes, while pathway analysis of RNA datasets pointed to a function of KLFs in behaviour and cognition. Possibly, complex mental processes such as cognition are influenced by upstream regulatory effects of MRs and GRs on the expression of genes that participate in neuronal processes, such as synaptic transmission.

Our study also provides evidence for a more widespread action of the MR in the brain than expected in view of early radioligand binding and *in vitro* autoradiography studies that detected hardly any MRs in the amygdala (except the central amygdala), PFC, and neocortex (Reul and De kloet, 1985, Reul and de Kloet, 1986; Reul et al., 1987). While our findings of MR ChIP-qPCR at *Klf* GREs do support the evidence that levels of MRs are highest in hippocampal regions, we detected comparable levels of MR binding to *Klf* GREs in the amygdala and substantial MR binding to *Klf* GREs in the 10.13039/501100000108PFC and neocortex, indicating a less restricted expression than previously thought. Therefore, although our studies do not demonstrate absolute expression levels, the immunoprecipitation of both receptors at *Klf* GREs does confirm their presence in these tissues.

Although mRNA levels of *Klf4* and *Klf9* have been shown to be GC sensitive (Datson et al., 2008; Reddy et al., 2009), only recently a more comprehensive approach addressing all Klf genes and their changes in activity under physiological conditions was conducted in MR and GR ChIP-seq and RNA-seq studies (Mifsud et al., 2021). The importance of establishing a link between expression of these *Klf* genes and GC hormone activity is reflected by the critical functions these transcription factors fulfil in the brain. *Klf9*, the first known *Klf* gene (Imataka et al., 1992), was initially identified as a thyroid responsive gene in the developing rat brain (Denver et al., 1999). The *Klf9* gene is expressed ubiquitously in tissues and, upon translation in the brain, KLF9 has been shown to play a role in motor learning and coordination (Morita et al., 2003), neurodevelopment (Scobie et al., 2009; Moore et al., 2009; Guo et al., 2022) and behavioural responses to stress (Besnard et al., 2018). Experiments involving *Klf9* knock-out (KO) mice highlighted the essential role of the gene in hippocampal (DG) dendritic spine differentiation, neuronal maturation, synaptic plasticity, anxiety-like behaviours, and learning (Scobie et al., 2009). Alongside several KLF family members, KLF9 is developmentally regulated during retinal ganglion cell growth, while overexpression of KLF9 significantly decreases axon regeneration following injury to the optic nerve (Moore et al., 2009). In the dentate gyrus of the hippocampus, an inverse relationship between *Klf9* expression and radial-glial neural stem cell (RGL) activation was reported, while RGLs assumed an activated state upon repression of *Klf9* (Guo et al., 2022).

The *Klf9* gene was identified as a GC receptor target gene at a relatively later stage. An increase in *Klf9* expression within the medial amygdala and other regions was reported in frogs exposed to shaking stress, while induced expression of KLF9 was shown to enhance neuronal differentiation and maturation reflected by increased neuronal density (Bonett et al., 2009). Acute restraint stress exposure has also been shown to increase hippocampal *Klf9* expression in mice, and genetic silencing of *Klf9* resulted in impaired behavioural responses to chronic stress and chronic CORT exposure in *Klf9* knock out mice (Besnard et al., 2018). The *Klf9* GRE identified by our ChIP-seq studies has also been described (Bagamasbad et al., 2012) in the mouse hippocampus, exhibiting 100% homology with the rat GRE. Mice injected postnatally with CORT showed increased *Klf9* hnRNA and mRNA levels, while treatment of mouse hippocampus-derived (HT-22) cell lines with CORT caused dose- and time-dependent increases in *Klf9* mRNA. In agreement with our study, CORT-dependent GR binding to GREs within *Klf9* was shown by ChIP and electrophoretic mobility shift assay (EMSA) in these cells. Interestingly, ChIP also showed a CORT-dependent increase in H3 acetylation at *Klf9* GREs (Bagamasbad et al., 2012) indicating MR and GR binding at these loci may be epigenetically regulated. Our FIMO analysis reveals an array of other prominent transcription factor binding sites such as early growth response (EGR) motifs, specificity protein (SP) motifs, cAMP-response element binding (CREB) motifs and KLF motifs. Thus, MRs and GRs may work via GREs in conjunction with other transcription factors as well as epigenetic modifications to regulate *Klf* gene expression and, in so doing, the biological processes in which they are involved.

*Klf2* plays a central role in the regulation of endothelial cells (ECs) within the blood-brain barrier (BBB) (Wu et al., 2013); a structure essential for healthy CNS functioning (Salvador et al., 2014). Many CNS disorders are accompanied by compromised integrity of the BBB, including stroke (Shi et al., 2013) and Alzheimer's disease (AD) (Fang et al., 2017). Notably, KLF2 has been shown to exert a neuroprotective effect following stroke by regulating the BBB in the cerebral cortex. Overexpression of KLF2 in mice led to smaller stroke volumes and protected against TNF-α-mediated BBB dysfunction (Shi et al., 2013). Decreased levels of *Klf2* mRNA and protein have been reported in both mouse models of AD (Wu et al., 2013) and in human post-mortem tissue of patients diagnosed with AD (Fang et al., 2017). We show here for the first time that the rat *Klf2* gene binds MR and GR, is stress-responsive and is regulated by GC hormones.

In contrast to *Klf9*, the role of *Klf1*5 has hardly been studied in the brain in relation to GC hormone activity. In this study, enrichment levels of MRs and GRs at *Klf1*5 GREs were slightly lower compared with levels seen at *Klf2* and *Klf9* GREs and, in contrast to the other *Klfs,* the induction of *Klf1*5 RNA did not exceed 2-fold in any of the brain regions investigated. *Klf15* expression in the rat brain has been shown to be relatively low in comparison to other tissues (Yu et al., 2014), possibly making it more difficult to detect, hence the relatively lower MR and GR enrichment and RNA expression observed. Our findings of expression and GC regulation of the *Klf15* gene in the brain, however, support a role of this 10.13039/501100016148KLF in the CNS.

RNA responses of the *Klf4* gene, which lacks MR and GR binding, were investigated to provide a negative control for studies involving MR and GR manipulation (adrenalectomy). Indeed, adrenalectomy did not affect BLAM and stress-induced *Klf4* mRNA levels. hnRNA levels of *Klf4* remained stable following exposure to stress, apart from a small increase in the PFC at FS30, while mRNA levels were increased at FS30 in all brain regions examined. It cannot be excluded that hnRNA levels of *Klf4* have risen prior to the FS30 timepoint due to rapidly acting epigenetic and/or transcription factors. Interestingly, the application of *N*-Methyl-*D*-aspartate (NMDA) to primary cortical neuron cultures induced a rapid upregulation of *Klf4* mRNA which was accompanied by upregulated KLF4 protein expression. Antagonism of the NMDA receptor by MK801 completely abolished the induction of *Klf4* mRNA expression, an effect that antagonists of AMPA or kainate receptors failed to produce (Zhu et al., 2009). Previously, we have shown that acute stress like novelty and FS, via the NMDA receptor, activates signalling and epigenetic pathways in the hippocampus (Chandramohan et al., 2007, 2008; Reul, 2014). Therefore, in our present study, it may be postulated that stress-induced Klf4 RNA expression in the hippocampus could be a result of NMDA receptor activation as well.

To further examine the role of GCs in the regulation of *Klf* gene expression following stress, we examined RNA levels in ADX rats. Previously (Kennedy et al., 2020), we showed that ADX abolished the FS-induced secretion of CORT as well as MR- and GR-binding to GREs and corresponding transcriptional responses in the GC target genes *Fkbp5, Per1* and *Sgk1.* Here, we report that ADX blocked the FS-induced rise of *Klf9* and *Klf1*5 hnRNA and mRNA levels and Klf2 hnRNA levels, whereas the effect on Klf2 mRNA was only partial*. Klf4* RNA responses were not affected by ADX underscoring its independence from GC action. These results show that stress-induced transcription of *Klf2*, *Klf9* and *Klf15* is critically dependent of GC action.

Our study assessed the genomic actions of CORT-activated MRs and GRs in brain regions of male rats only. As we intended this research to be a follow up from our genome-wide MR and GR ChIP-seq and RNA-seq studies on the male rat hippocampus (Mifsud et al., 2021), inclusion of female rats could confuse this study as no comparable ChIP- and RNA-seq has been done yet. Such sequencing studies are certainly important given the effects of the estrus cycle on chromatin dynamics (Rocks et al., 2022). Chromatin architecture is a major determining factor in the accessibility of DNA sequences for binding of transcription factors such as MRs and GRs, and densely packed chromatin can repress transcription by impeding protein-DNA interactions (King et al., 2012). Currently, we are conducting sequencing studies aiming to address sex- and cycle-specific changes in MR and GR interaction with the hippocampus genome under baseline and stress conditions.

KLFs comprise an important family of transcription factors that regulate functions like neuronal differentiation and development in the brain. Here, we demonstrated that, in several brain regions, specific *Klfs* are target genes of genomically acting MRs and GRs following physiological rises in circulating CORT levels. This work further substantiates a role of KLFs in the molecular effects of GCs in relation to stress adaptation and memory formation. Our work also suggests that, in addition to MRs and GRs, other transcription factors including KLFs as well as other (e.g. epigenetic) mechanisms may play a role in the response of Klf genes to physiological changes like acute stress and circadian variation. Elucidation of these participating mechanisms should be an interesting avenue of future research.

## Credit author statement

CLMK, EMP, KRM and JMHMR conceptualized and designed the study. CLMK, EMP, KRM, HMG and JMHMR conducted the animal experiments. CLMK, HMG and KRM conducted ChIP, RNA, and qPCR analyses and analysed the data. CLMK and JMHMR wrote the manuscript. EMP contributed to the writing of the manuscript. SS and ES processed the next-generation sequencing (NGS) data and conducted extensive bioinformatics analysis of these data. JB conducted the quality control. SE coordinated the library preparation and the next-generation sequencing (NGS) of the ChIP DNA and RNA samples.

## Declaration of competing interest

The authors declare that they have no known competing financial interests or personal relationships that could have appeared to influence the work reported in this paper.

## Data Availability

The ChIP- and RNA-sequencing data are accessible through GEO Series accession number GSE126706: https://www.ncbi.nlm.nih.gov/geo/query/acc.cgi?acc=GSE126706.

## Appendix A Supplementary data

**Supplementary Fig. 1. Hippocampal MR and GR ChIP-seq shows binding of MRs and GRs at GREs within *Klf2, Klf9* and *Klf15* under baseline and stressed conditions.**Rats were killed under early morning (BLAM) or late afternoon (BLPM) baseline conditions or 30 min (FS30) after the start of FS (15 min, 25 °C water). Graphs show enrichment of MR and GR at GREs within *Klf2* (A and B), *Klf9* (C and D) and *Klf15* (E − H) which was calculated by Differential binding (DiffBind) analysis. ***P* < 0.01, ****P* < 0.001, *****P* < 0.0001 significantly different from BLAM.**Supplementary Fig. 2. Hippocampal inRNA and exRNA transcript counts of KLFs under baseline conditions and following stress.**Rats were killed under early morning (BLAM) or late afternoon (BLPM) baseline conditions, or 30 min (FS30), 60 min (FS60), 120 min (FS120), 180 min (FS180) or 360 min (FS360) after the start of FS (15 min, 25 °C water. Graphs show inRNA (blue bars) and exRNA (red bars) counts of *Klf2* (A), *Klf4* (B) *Klf9* (C) and *Klf15* (D). Data are shown as percentage of transcripts per million at BLAM. Differential expression analysis, **P* < 0.05, significantly different from BLAM.**Supplementary Fig. 3. PFC MR and GR binding at GREs within *Klf2, Klf9* and *Klf15* under baseline and stressed conditions.**Rats were killed under early morning (BLAM) or late afternoon (BLPM) baseline conditions or 30 min (FS30) after the start of FS (15 min, 25 °C water). Graphs show enrichment of MR and GR, expressed as bound/input (B/I; mean ± SEM, n = 4 per group), at GREs within *Klf2* (A and B), *Klf9* (C and D), *Klf15* (E-H) as determined by MR and GR ChIP-qPCR. Statistical analysis; One-way ANOVA (A) F_(2, 9)_ = 14.63, *p* = 0.0015, (B) F_(2, 9)_ = 17.82, *p* = 0.0007, (C) F_(2, 9)_ = 9.935, *p* = 0.0053, (D) F_(2, 9)_ = 11.84, *p* = 0.0030, (E) F_(2, 9)_ = 14.57, *p* = 0.0015, (F) F_(2, 9)_ = 9.89, *p* = 0.00531, (G) F _(2, 9)_ = 20.33, *p* = 0.0005, (H) F_(2, 9)_ = 18.46, *p* = 0.0007. Dunnett's post hoc test, **P* < 0.05 ***P* < 0.01, ****P* < 0.001 significantly different from BLAM.**Supplementary Fig. 4. Neocortex MR and GR binding at GREs within Klf2, Klf9 and Klf15 under baseline and stressed conditions.**Rats were killed under early morning (BLAM) or late afternoon (BLPM) baseline conditions or 30 min (FS30) after the start of FS (15 min, 25 °C water). Graphs show enrichment of MR and GR, expressed as bound/input (mean ± SEM, n = 4 per group), at GREs within Klf2 (A and B), Klf9 (C and D), Klf15 (E-H) as determined by MR and GR ChIP-qPCR. Statistical analysis; One-way ANOVA (A) F_(2, 9)_ = 10.07, *p* = 0.0051, (B) F_(2, 9)_ = 10.49, *p* = 0.0045, (C) F_(2, 9)_ = 7.987, *p* = 0.0101, (D) F_(2, 9)_ = 5.788, *p* = 0.0242. (E) F_(2, 9)_ = 5.267, *p* = 0.0306, (F) F _(2, 9)_ = 6.535, *p* = 0.0177, (G) F _(2, 9)_ = 3.71, *p* = 0.066, (H) F_(2, 9)_ = 4.49, *p* < 0.0444. Dunnett's post hoc test, **P* < 0.05, ***P* < 0.01 significantly different from BLAM.**Supplementary Fig. 5. PFC hnRNA and mRNA of KLFs under baseline conditions and following stress.**Rats were killed under early morning (BLAM) or late afternoon (BLPM) baseline conditions or 30 min (FS30), 60 min (FS60) or 120 min (FS120) min after the start of FS (15 min, 25 °C water). Graphs show hnRNA (blue bars) and mRNA (red bars) levels of *Klf2* (A), *Klf4* (B) *Klf9* (C) and *Klf15* (D). Data are shown as relative RNA copy number calculated using the Pfaffl method of analysis, standardised to the expression of the house keeping genes *Hprt1* and *Ywhaz* (mean ± SEM, n = 8–9 per group). Statistical analysis: one-way ANOVA; (A) *Klf2* hnRNA F_(4, 40)_ = 24.53, *p* < 0.0001, *Klf2* mRNA F_(4, 40)_ = 113.1, *p* < 0.0001, (B) *Klf4* hnRNA F_(4, 40)_ = 6.373, *p* < 0.001, *Klf4* mRNA F_(4, 40)_ = 62.04, *p* < 0.0001, (C) *Klf9* hnRNA F_(4, 40)_ = 27.81, *p* < 0.0001, *Klf9* mRNA F_(4, 40)_ = 12.88, *p* < 0.0001, (D) *Klf1*5 hnRNA F_(4, 40)_ = 18.54, *p* < 0.0001, *Klf1*5 mRNA F_(4, 40)_ = 22.03, *p* < 0.0001. Dunnett's post hoc test, **P* < 0.05, ***P* < 0.01, ****P* < 0.001 significantly different from BLAM.**Supplementary Fig. 6. Neocortex hnRNA and mRNA of KLFs under baseline conditions and following stress.**Rats were killed under early morning (BLAM) or late afternoon (BLPM) baseline conditions or 30 min (FS30), 60 min (FS60) or 120 min (FS120) min after the start of FS (15 min, 25 °C water). Graphs show hnRNA (blue bars) and mRNA (red bars) levels of *Klf2* (A), *Klf4* (B) *Klf9* (C) and *Klf15* (D). Data are shown as relative RNA copy number calculated using the Pfaffl method of analysis, standardised to the expression of the house keeping genes *Rpl10a* and *Cyclophilin A* (mean ± SEM, n = 8–9 per group). Statistical analysis: one-way ANOVA; (A) *Klf2* hnRNA F_(4, 39)_ = 37.63, *p* < 0.0001, *Klf2* mRNA F_(4, 39)_ = 119.4, *p* < 0.0001, (B) *Klf4* hnRNA F_(4, 40)_ = 3.659, *p* = 0.0124, *Klf4* mRNA F_(4, 37)_ = 17.19, <0.0001, (C) *Klf9* hnRNA F_(4, 39)_ = 53.45, *p* < 0.0001, *Klf9* mRNA F_(4, 40)_ = 6.177, *p* < 0.001, (D) *Klf1*5 hnRNA F_(4, 39)_ = 38.56, *p* < 0.0001, *Klf1*5 mRNA F_(4, 40)_ = 6.2606, *p* = 0.0006. Dunnett's post hoc test, **P* < 0.05, ***P* < 0.01, ****P* < 0.001 significantly different from BLAM.
