# Supplementary material for: Genomic regulation of Krüppel-like-factor family members by corticosteroid receptors in the rat brain
Source: Neurobiol Stress. 2023 Mar 7;23:100532. doi: 10.1016/j.ynstr.2023.100532 (PMC10024234; doi:10.1016/j.ynstr.2023.100532)
Supplement: Multimedia component 7 [file mmc7.docx]

**Supplementary Table 7. Upstream regulator analysis predicts KLF transcription factor family members as regulators of GC target genes**

| **Upstream regulator** | **Dataset** | **Predicted Activation**  **state** | **Activation z-score** | **p-value**  **of overlap** | **# Target genes in dataset** |
| --- | --- | --- | --- | --- | --- |
| **KLF1** | inRNA | N/A | N/A | 0.0476 | 7 |
| **KLF2** | inRNA | N/A | 1.219 | 0.018 | 19 |
|  | exRNA | N/A | -0.831 | 2.57e-07 | 13 |
| **KLF3** | GR | N/A | -1.739 | 0.043 | 17 |
|  | exRNA | Inhibited | -2.345 | 0.0455 | 9 |
| **KLF4** | inRNA | N/A | -1.366 | 6.62e-03 | 31 |
|  | exRNA | N/A | 1.639 | 5.27e-06 | 15 |
| **KLF5** | GR | N/A | 1.070 | 7.34e-03 | 6 |
|  | exRNA | N/A | N/A | 0.049 | 3 |
| **KLF6** | MR | N/A | -0.392 | 0.0491 | 8 |
|  | GR | N/A | 0.513 | 0.0275 | 6 |
|  | inRNA | N/A | -0.417 | 4.99e-03 | 12 |
|  | exRNA | N/A | -0.77 | 6.05e-07 | 9 |
| **KLF10** | exRNA | N/A | N/A | 0.0228 | 2 |
| **KLF15** | exRNA | N/A | N/A | 0.0232 | 3 |
| **KLF16** | exRNA | N/A | N/A | 0.0411 | 1 |

Datasets generated by ChIP- and RNA-sequencing experiments were input to Ingenuity Pathway Analysis (IPA) software. A “core” analysis was performed on the genes annotated to MR and GR peaks (ChIP-seq) and genes exhibiting differential RNA expression (RNA-seq) following acute stress and during the circadian rise. Pathway analysis predicted that KLFs behave as upstream regulators of genes in the ChIP-seq and RNA-seq datasets. Activation z-scores ≥ +2 significantly predict pathway activation, while z-scores of ≤ -2 significantly predict pathway inhibition. Activation z-scores < +2 or > -2 indicate a trend towards activation or inhibition, however the prediction is not statistically significant. N/A indicates that a predicted activation state or an activation z-score was not calculated by IPA. Statistical analysis: Right-tailed Fisher’s exact test, p<0.05.
