# Supplementary material for: Genomic regulation of Krüppel-like-factor family members by corticosteroid receptors in the rat brain
Source: Neurobiol Stress. 2023 Mar 7;23:100532. doi: 10.1016/j.ynstr.2023.100532 (PMC10024234; doi:10.1016/j.ynstr.2023.100532)
Supplement: Multimedia component 8 [file mmc8.docx]

**Supplementary Table 8. Pathway analysis predicts KLF transcription factor family members to play a role in neurodevelopmental pathways and behaviours**

| **KLF** | **Dataset** | **Annotation** | **p-value** | **Predicted Activation State** | **Activation z-score** | **# Other Molecules** |
| --- | --- | --- | --- | --- | --- | --- |
| **KLF9** | MR, GR, exRNA | Development of neurons | 8.67e-06 | Increased | 3.26 | 106 |
| **KLF9** | MR, GR | Microtubule dynamics | 0.000061 | Increased | 3.063 | 100 |
| **KLF9** | MR, GR | Maturation of neurons | 0.000492 | Increased | 2.414 | 14 |
| **KLF9** | MR, GR | Branching of neurons | 0.00254 | Increased | 2.576 | 45 |
| **KLF9** | MR, GR | Plasticity of synapse | 0.00287 |  | 1.756 | 20 |
| **KLF9** | MR | Shape change of neurites | 0.00468 | Increased | 3.238 | 44 |
| **KLF9** | MR, GR | Dendritic growth/branching | 0.0292 | Increased | 2.71 | 20 |
| **KLF15** | MR, GR | Differentiation of neuroglia | 0.0475 | Increased | 2.789 | 13 |
| **KLF9** | inRNA | Anxiety | 1.22E-07 | N/A | 1.425 | 44 |
| **KLF9** | inRNA, exRNA | Cognition | 3.13E-12 | N/A | 0.224 | 98 |
| **KLF9** | inRNA, exRNA | Learning | 2.23E-10 | N/A | 0.178 | 87 |

Datasets comprising genes annotated to MR and GR peaks and genes exhibiting differential RNA expression following acute stress or during the circadian rise were analysed by IPA. Core analysis predicted KLFs to participate in biological functions and diseases, of which a subset of neurobiological pathways and behaviours are highlighted above. The total number of GC target genes predicted to participate in the biological pathway is shown. Activation z-scores ≥ +2 significantly predict pathway activation, while z-scores of ≤ -2 significantly predict pathway inhibition. Activation z-scores < +2 or > -2 indicate a trend towards activation or inhibition, however the prediction is not statistically significant. N/A indicates that a predicted activation state or an activation z-score was not calculated by IPA. Statistical analysis: Right-tailed Fisher’s exact test, p<0.05. For pathways predicted by analysis of multiple datasets, p-values and activation z-scores from the MR analysis or the inRNA analysis is displayed. All values and details of GC target genes can be found in the supplemental tables.
