# Supplementary material for: Genomic regulation of Krüppel-like-factor family members by corticosteroid receptors in the rat brain
Source: Neurobiol Stress. 2023 Mar 7;23:100532. doi: 10.1016/j.ynstr.2023.100532 (PMC10024234; doi:10.1016/j.ynstr.2023.100532)
Supplement: Multimedia component 9 [file mmc9.docx]

**Supplementary Figure 1**
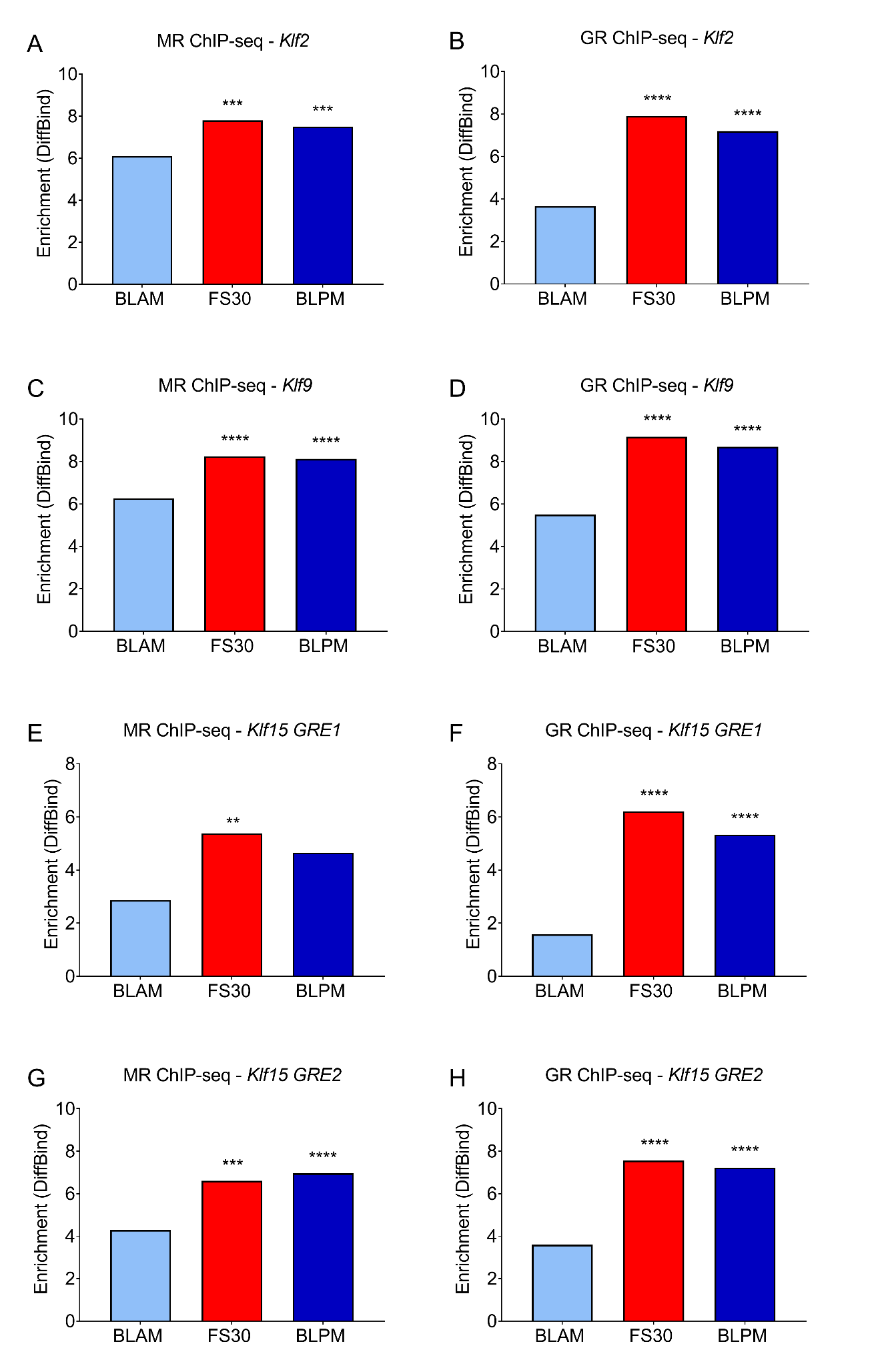


**Supplementary Figure 2**


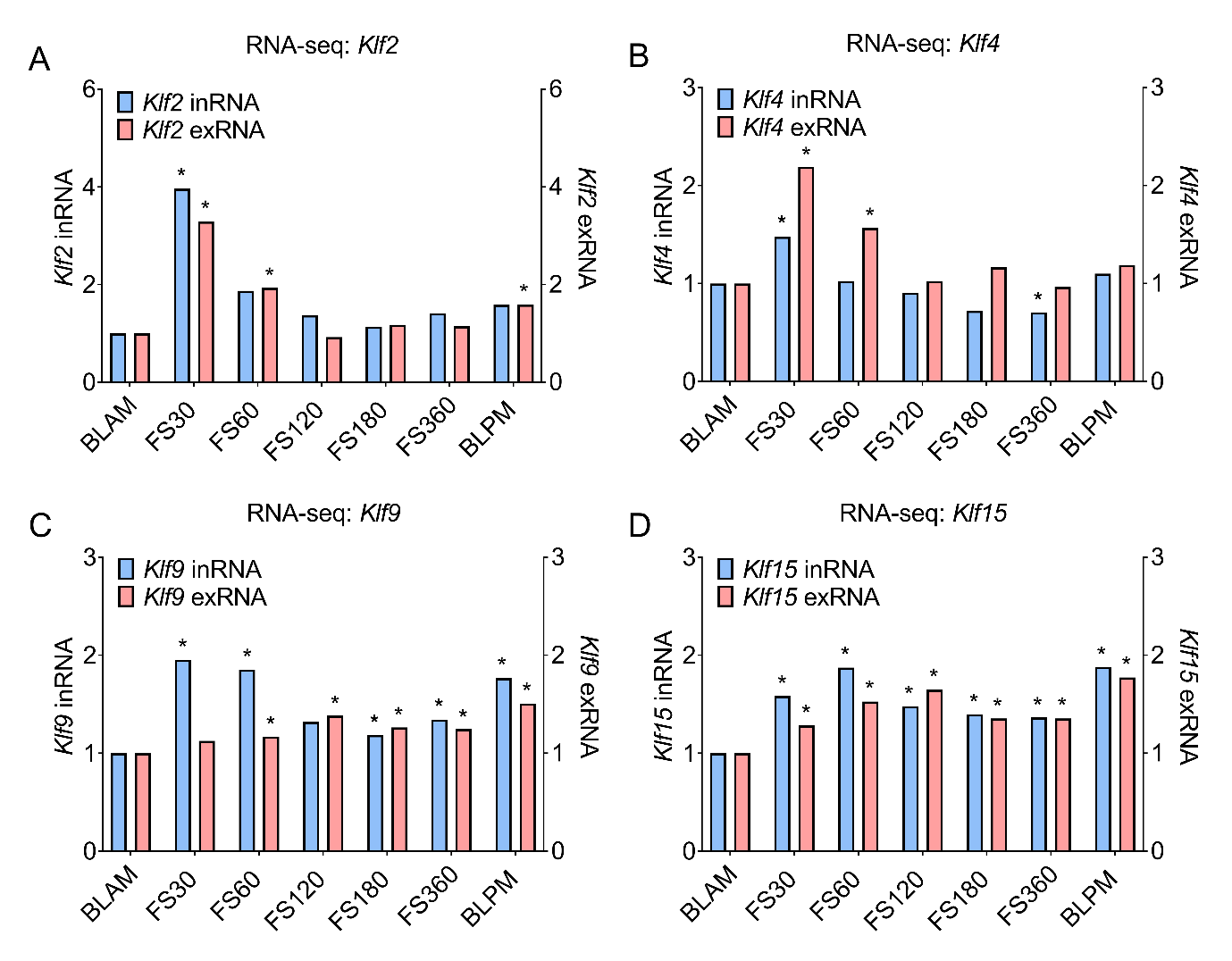


**Supplementary Figure 3**


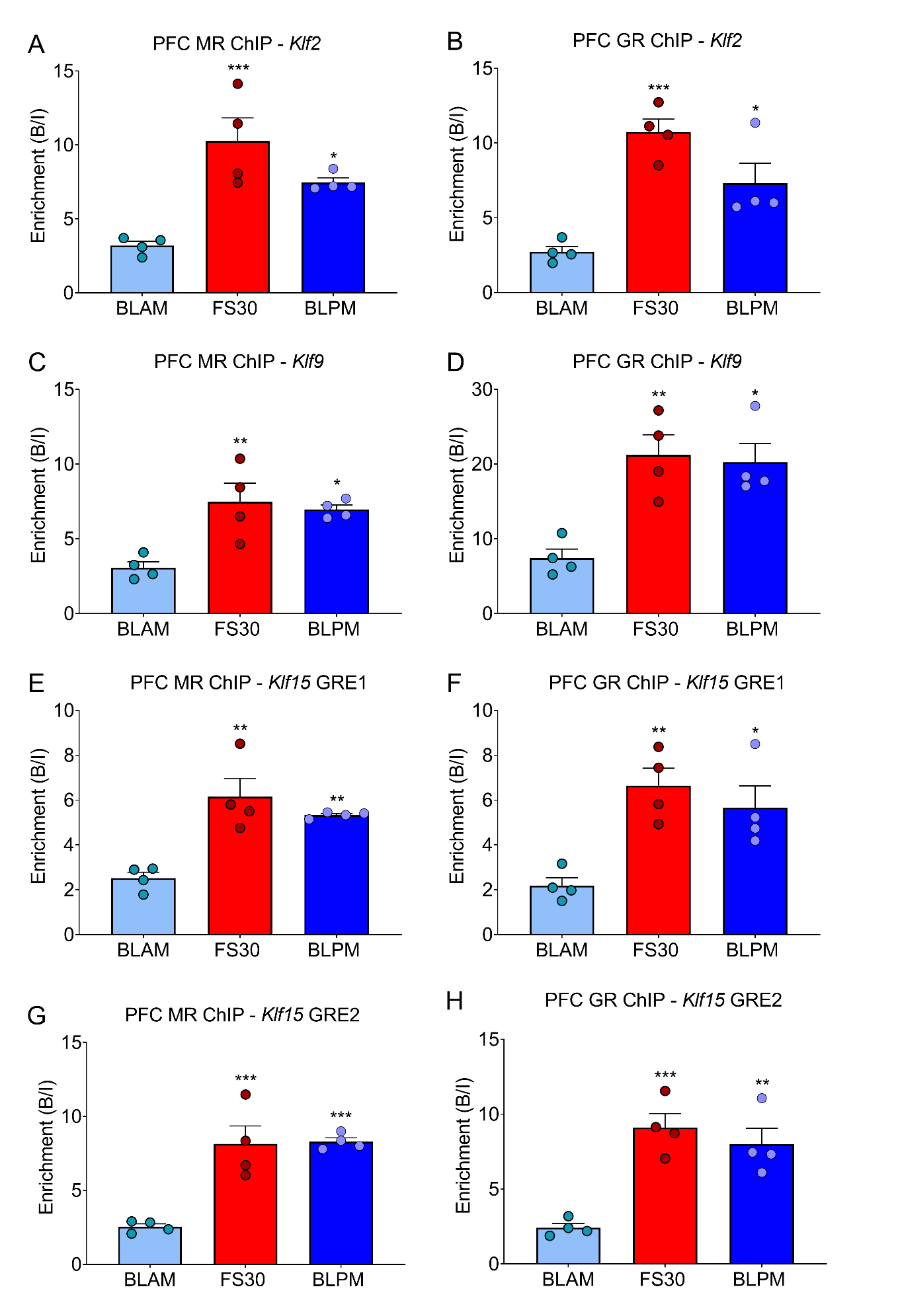


**Supplementary Figure 4**


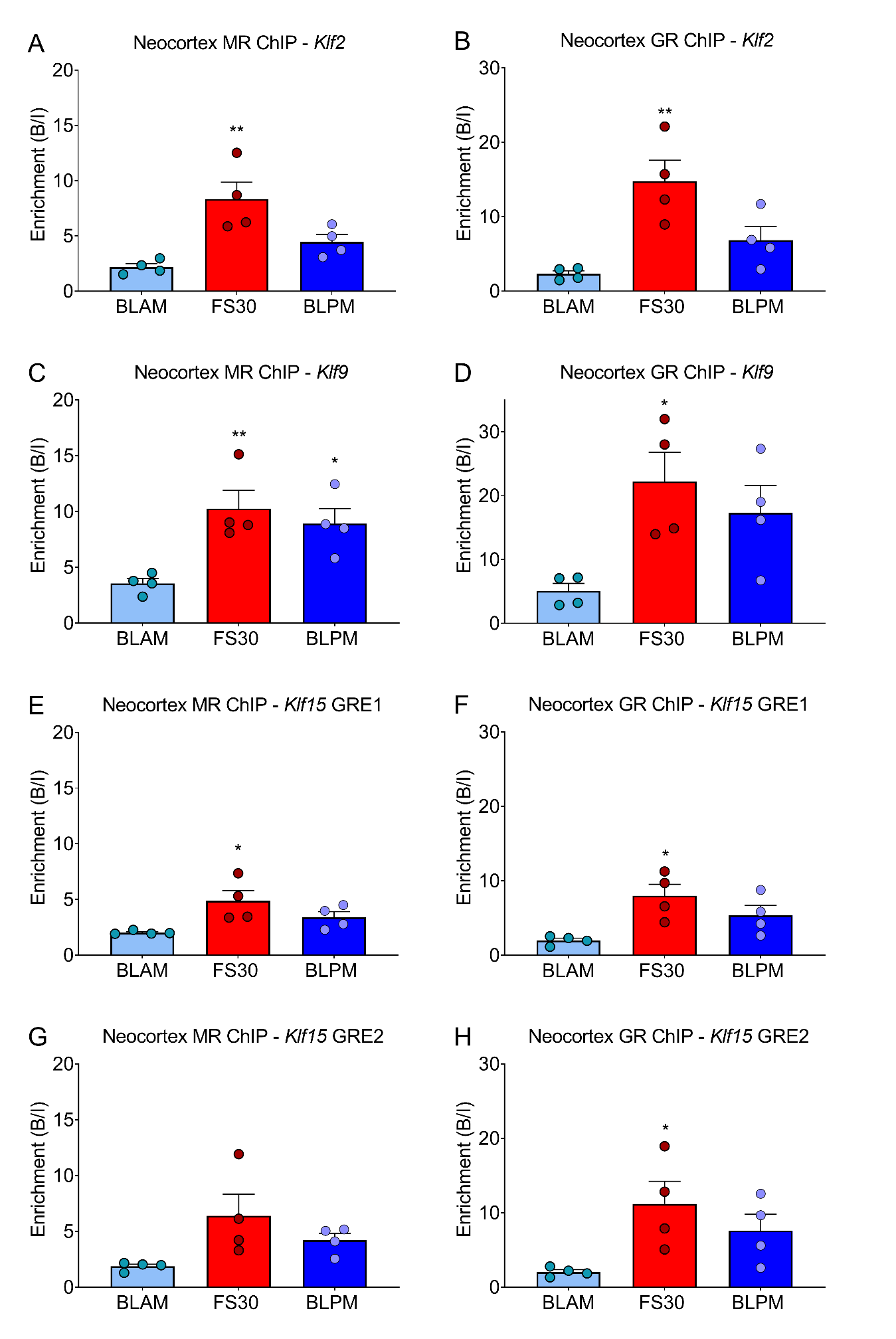


**Supplementary Figure 5**


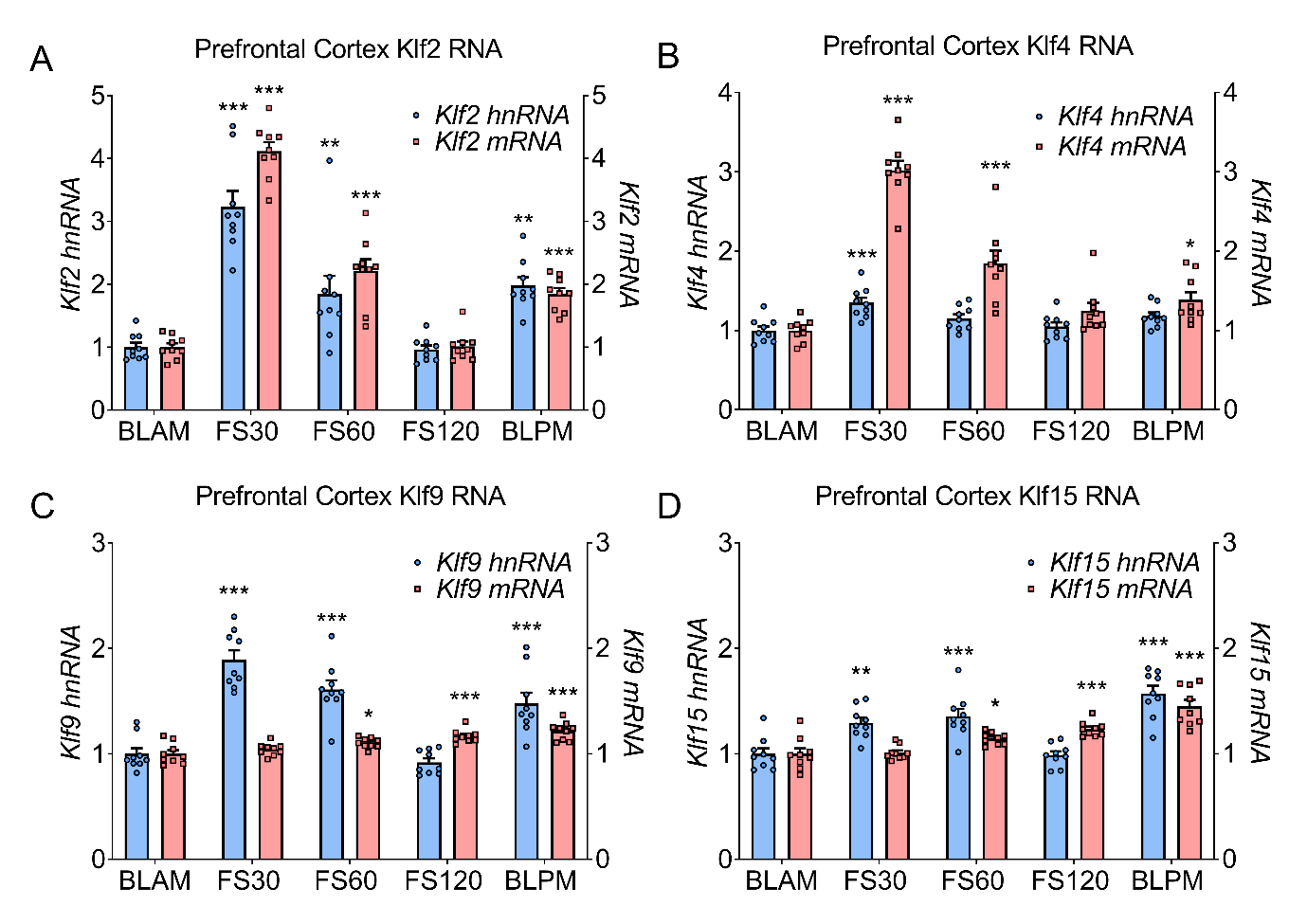


**Supplementary Figure 6**


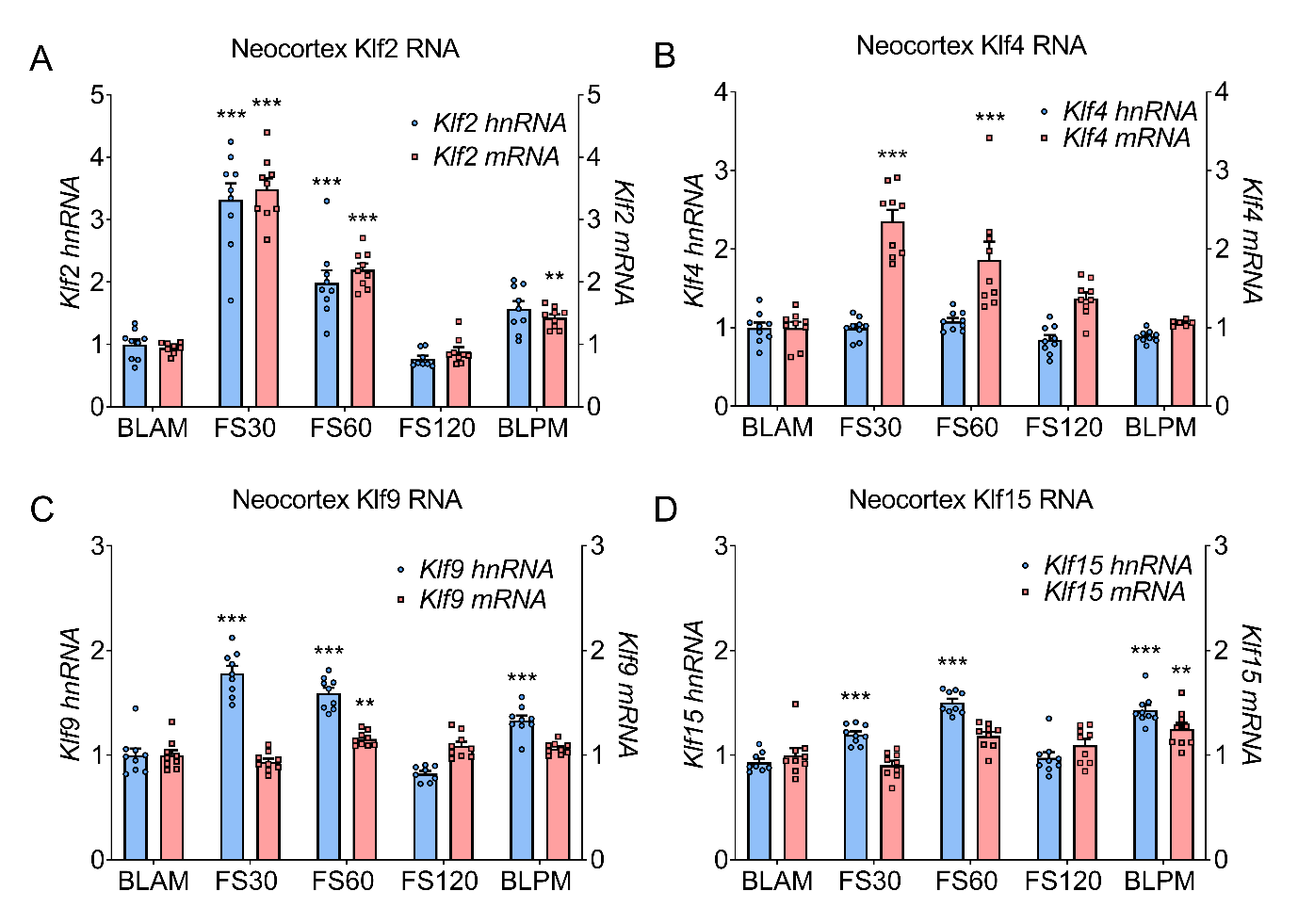


**List of Supplementary Tables**

1. ChIP primers and probes for *Klf* genes.
2. RNA primers and probes.
3. House-keeping gene primers and probes.
4. MR and GR peak coordinates associated with *Klf2*, *Klf9* and *Klf15* genes.
5. FIMO analysis of MR peaks associated with *Klf* genes.
6. FIMO analysis of GR peaks associated with *Klf* genes.
7. *Klf* genes as upstream regulators as determined by IPA analysis.
8. IPA pathway analysis of *Klf* genes.
